# Testing Behavioral Messages to Increase Recruitment to Health Research When Embedded Within Social Media Campaigns on Twitter: Web-Based Experimental Study

**DOI:** 10.2196/48538

**Published:** 2024-02-05

**Authors:** Sandro T Stoffel, Jing Hui Law, Robert Kerrison, Hannah R Brewer, James M Flanagan, Yasemin Hirst

**Affiliations:** 1 Department of Behavioural Science and Health University College London London United Kingdom; 2 Institute of Pharmaceutical Medicine University of Basel Basel Switzerland; 3 Wolfson Institute of Population Health Queen Mary University of London London United Kingdom; 4 School of Health Sciences University of Surrey Guildford United Kingdom; 5 Department of Surgery and Cancer Imperial College London London United Kingdom; 6 Lancaster Medical School Lancaster University Lancaster United Kingdom; 7 Applied Health Research Hub University of Central Lancashire Preston United Kingdom

**Keywords:** advertise, advertisement, advertisements, advertising, behavior change, behavioral, behaviour change, behavioural, campaign, campaigns, experimental design, message, messages, messaging, recruit, recruiting, recruitment, social media, social norms, Twitter

## Abstract

**Background:**

Social media is rapidly becoming the primary source to disseminate invitations to the public to consider taking part in research studies. There is, however, little information on how the contents of the advertisement can be communicated to facilitate engagement and subsequently promote intentions to participate in research.

**Objective:**

This paper describes an experimental study that tested different behavioral messages for recruiting study participants for a real-life observational case-control study.

**Methods:**

We included 1060 women in a web-based experiment and randomized them to 1 of 3 experimental conditions: standard advertisement (n=360), patient endorsement advertisement (n=345), and social norms advertisement (n=355). After seeing 1 of the 3 advertisements, participants were asked to state (1) their intention to take part in the advertised case-control study, (2) the ease of understanding the message and study aims, and (3) their willingness to be redirected to the website of the case-control study after completing the survey. Individuals were further asked to suggest ways to improve the messages. Intentions were compared between groups using ordinal logistic regression, reported in percentages, adjusted odds ratio (aOR), and 95% CIs.

**Results:**

Those who were in the patient endorsement and social norms–based advertisement groups had significantly lower intentions to take part in the advertised study compared with those in the standard advertisement group (aOR 0.73, 95% CI 0.55-0.97; *P*=.03 and aOR 0.69, 95% CI 0.52-0.92; *P*=.009, respectively). The patient endorsement advertisement was perceived to be more difficult to understand (aOR 0.65, 95% CI 0.48-0.87; *P*=.004) and to communicate the study aims less clearly (aOR 0.72, 95% CI 0.55-0.95; *P*=.01). While the patient endorsement advertisement had no impact on intention to visit the main study website, the social norms advertisement decreased willingness compared with the standard advertisement group (157/355, 44.2% vs 191/360, 53.1%; aOR 0.74, 95% CI 0.54-0.99; *P*=.02). The majority of participants (395/609, 64.8%) stated that the messages did not require changes, but some preferred clearer (75/609, 12.3%) and shorter (59/609, 9.7%) messages.

**Conclusions:**

The results of this study indicate that adding normative behavioral messages to simulated tweets decreased participant intention to take part in our web-based case-control study, as this made the tweet harder to understand. This suggests that simple messages should be used for participant recruitment through Twitter (subsequently rebranded X).

## Introduction

For researchers, increasing use of the internet has opened up new ways to investigate society and behavior at lower costs [1], producing higher data quality (Kongsved et al [2]), faster return rates, and lower data entry times [3]. Web-based participant recruitment (eg, websites and apps, such as social media) has also been applied in the social and behavioral sciences.

The new internet, or social media, also known as Web 2.0, enables people to connect with friends, family, companies, and other entities to produce and share content on the web [4,5]. The benefits of using social media for participant recruitment, over and above Internet 1.0, have been increasingly demonstrated [6,7]. For example, at the height of the COVID-19 pandemic in 2020, social media allowed many social scientists to connect with thousands of people and include populations with unmet needs in their research [8-10]. In addition, compared to paid panels, using paid social media advertisements to recruit research participants has been shown to cost less [11,12] and be more time-efficient [13,14]. Furthermore, it is also suggested that social media can reach larger pools of participants and access hard-to-reach populations [15]. Social media allow researchers to recruit participants quickly and cost-effectively, as they are accessible through various devices at any time and allow the creation of specific digital content to target specific populations, increasing the likelihood of achieving the required sample size [15,16].

Facebook, Twitter (subsequently rebranded X), and Instagram are some of the most popular social media platforms for health research [13,17-20]. In 2022, more than 70% of internet users in the United Kingdom reported using Facebook and 42.8% Twitter [21]. Social media has become so deeply embedded in our daily lives that people rely on them for every need, such as entertainment, information, purchases, social connections, and work [22]. As new social media platforms enter the market, it is expected that the number of social media users will continue to grow [22].

So far, only a few studies have investigated the effectiveness of social media for health research. Most studies have investigated their effectiveness concerning recruitment in offline studies [13,16,17,23], with only a few making comparisons between different web-based recruitment methods for web-based studies [24]. While there is some guidance for the ethical use of social media for recruiting participants for health research [25], little is known about optimizing the use of platforms such as Twitter and Facebook for health-related research recruitment [19,26], such as the contents of the message to be used for targeting eligible individuals.

However, we can infer potential key components of a social media message from other areas using behavioral sciences. For example, marketing research has shown that credibility and trust in the source are important factors for clicking on advertisements [17,27-30]. In relation to this, studies have suggested that web-based health information from an expert source is viewed as more experienced and credible [31,32]. These findings demonstrate that aspects of endorsement and credibility when creating and disseminating messages are important. Similarly, several studies have shown that messages containing descriptive and normative social norms can be effective methods of engaging with the public [33-35]. In these studies, individuals receive information about socially desired (normative norms) or most frequently observed behavior (descriptive norms). Social norm messages provide individuals with a standard against which they can compare their intentions [36]. To our knowledge, no previous studies have tested whether social norms or patient endorsement messages on social media posts increase engagement with target audiences. The primary aim of this web-based experimental study, therefore, was to design and test the use of tailored Twitter posts, which integrate elements of patient endorsement and social norms, for the recruitment of participants into an observational case-control study.

## Methods

### Setting and Context

In 2020, a simulated randomized web-based experiment was programed on SurveyMonkey (SurveyMonkey Inc). The experiment was designed to test the effectiveness of targeted social media messages to increase intentions to participate in a real-world observational case-control study called the Cancer Loyalty Card Study (CLOCS) [37]. CLOCS is an observational case-control study that aims to investigate the self-care behaviors of patients with ovarian cancer before their cancer diagnosis. It seeks to do this by investigating differences in transactional data (such as medication purchasing) between women with and without ovarian cancer (the transactional data are collected through the loyalty cards of 2 UK-based high street retailers). Cases (ie, women with ovarian cancer) were recruited through participating National Health Service sites, while controls were recruited through the study website. Thus, those who were eligible to take part as control participants were recruited through social media and other internet-based sources.

### Study Eligibility and Recruitment

The study sample comprised women aged between 18 and 70 years living in the United Kingdom without an ovarian cancer diagnosis who were potentially eligible for the real-world observational case-control study. Study participants were recruited through a web-based survey vendor, Dynata (Dynata Global UK Ltd).

### Procedure

At the beginning of the experiment, those who were interested in taking part in the web-based experiment were presented with information about the study, including a brief description of CLOCS as well as a consent form. If participants consented and were eligible, they were randomized (in a 1:1:1 ratio) to receive 1 of 3 simulated Twitter posts: a standard advertisement (control condition), an advertisement with patient endorsement (patient endorsement condition), or an advertisement with a descriptive social norms message (social norms condition; Table 1). To generate authentic Twitter messages, real tweets were posted on a dummy Twitter account, alongside an infographic detailing information about CLOCS. Screenshots were taken of these posts for use in the experiment (the messages were immediately deleted after each one was posted; Figures S1, S2, and S3 in Multimedia Appendix 1 contain screenshots of the messages).

**Table 1:** Messages used in the experimental study with readability scores and character count. The Flesch-Kincaid readability score ranges from 0 (“extremely difficult to read, best understood by university graduates”) to 100 (“very easy to read, easily understood by an average 11-year-old student”).

Condition	Content	Readability score without special symbols or URL	Readability score with special symbols and URL	Number of characters with special symbols or URL
Control condition	**NEW RESEARCH RECRUITING WOMEN WITHOUT #OVARIANCANCER** We are recruiting women in the UK, aged 18+ without #ovariancancer to an online survey about potential symptoms, shopping and self-care behaviours. Take part @ clocsproject.org.uk/participants	23.8	15	222
Patient endorsement condition	**NEW RESEARCH RECRUITING WOMEN WITHOUT #OVARIANCANCER** Fiona (CLOCS patient representative): #ParticipatedinCLOCS because I bought medication for my symptoms from retailers before my cancer diagnosis.” Take part @ clocsproject.org.uk/participants	24	7.9	224
Descriptive norms condition	**NEW RESEARCH RECRUITING WOMEN WITHOUT #OVARIANCANCER** Most women with #ovariancancer are happy to take part in CLOCS. You can help us better understand their illness and symptoms by taking part as a healthy volunteer. Take part @ clocsproject.org.uk/participants	57.8	37	232

The content of the messages is presented in Table 1, along with a Flesch-Kincaid readability score calculated using the web-based software Grammarly (Max Lytvyn, Dmytro Lider, and Alex Shevchenko). This was done to ensure that the message was understandable to the target audience [38]. The standard and patient endorsement messages had the lowest readability scores (15 and 7.9, respectively) and were the easiest to understand (Table 1).

After being presented with the Twitter messages, participants were asked 2 comprehension questions on whether CLOCS only recruits women with ovarian cancer and what kind of data CLOCS are analyzing. Participants could only continue in the survey if they answered the questions correctly [35,39,40]. The primary outcome was participants’ intention to take part in CLOCS, and we asked individuals whether they would participate in the advertised study, adapted from previous literature [34,35,39,41,42]. It featured a fully labeled 4-point response scale (“definitely not,” “probably not,” “yes probably,” and “yes definitely”).

To explore how the messages were perceived by the participants, we included 2 questions on how easy the message was to understand (“very difficult,” “fairly difficult,” “fairly easy,” or “very easy”) and how clearly the aims of the study were communicated (“not at all,” “a little,” “very,” or “extremely”).

In the next step, participants were asked about their past participation in health care research (“yes” or “no”) and whether they had loyalty cards from UK-based high-street retailers. Sociodemographic questions covered age (“18-24,” “25-34,” “35-44,” “45-54,” or “55-70”), education (“no college degree” or “college degree, equivalent, or higher”), employment status (“yes” or “no”), marital status (“single”, “married or living with a partner”, “divorced or separated or widowed”), self-reported health (“poor,” “fair,” “good,” or “excellent”), and history of cancer in themselves, family, or close friends (“yes” or “no”).

Individuals were then given the opportunity to state their thoughts on improving social media messages for the recruitment of study participants in an open-ended question.

The survey concluded with an active interest question on whether participants would be interested in being redirected to the CLOCS website for more information on how to participate [34,40-42]. Those who responded yes were provided with a link to the CLOCS website on the final page of the survey. The website opened in a new tab for participants who clicked on the link. No further data associated with their direct participation in CLOCS were collected in this experiment. The web-based experiment took, on average, 5 minutes to complete.

### Ethical Considerations

Ethics approval for this study was obtained from the University College London Research Ethics Committee (17813/001). All participants provided consent to take part in the study. All the data collected as part of the study were anonymized, meaning no identifiable information were collected. Eligible participants who completed the questionnaire received a small financial incentive from Dynata, as per their panelist agreements.

### Data Analysis

A pilot study was conducted beforehand for sample size calculations. Based on the findings from the initial sample of 359 participants, with a 10 percentage point difference in the intention to take part (“yes, definitely” or “yes, probably” versus “definitely no” or “probably no”), we determined that the number of participants needed to achieve 95% CI and 80% power was 350 per trial arm. Data from participants in both the pilot and final samples were combined for analysis.

Sample characteristics were assessed using descriptive statistics (Table 2). Differences in participants’ intention to take part in CLOCS and perception of the messages were assessed using univariate and multivariate ordinal logistic regression. Willingness to visit the actual website was assessed between groups using univariate and multivariate binary logistic regressions. Adjusted odds ratios (aORs), 95% CIs, and *P* values are presented in the results, with *P* values below .05 regarded as statistically significant.

The responses to the open-ended feedback question were categorized into main themes through content analysis [43].

**Table 2:** Sociodemographic characteristics of study participants.

Demographic categories						Control condition (n=360), n (%)		Patient endorsement condition (n=345), n (%)		Social norms condition (n=355), n (%)		Overall (N=1060), n (%)		Chi-square test (*df*)			*P* value^a^	
**Age group (years)**															8.54 (8)			.38
	18-24					48 (13.3)		45 (13.0)		51 (14.4)		144 (13.6)						
	25-34					71 (19.7)		74 (21.4)		76 (21.4)		221 (20.8)						
	35-44					80 (22.2)		87 (25.2)		86 (24.2)		253 (23.9)						
	45-54					76 (21.1)		74 (21.4)		87 (24.5)		237 (22.4)						
	55-70					85 (23.6)		65 (18.8)		55 (15.5)		205 (19.3)						
**Health**															12.40 (6)			.05
	Poor					15 (4.2)		17 (4.9)		25 (7.0)		57 (5.4)						
	Fair					94 (26.1)		118 (34.2)		90 (25.4)		302 (28.5)						
	Good					201 (55.8)		175 (50.7)		190 (53.5)		566 (53.4)						
	Excellent					50 (13.9)		35 (10.1)		50 (14.1)		135 (12.7)						
**Education**															4.67 (2)			10
	Lower than a college degree					171 (47.5)		181 (52.5)		197 (55.5)		549 (51.2)						
	College degree, equivalent, or higher					189 (52.5)		164 (47.5)		158 (44.5)		511 (48.2)						
**Paid employment**															0.09 (2)			.96
			Yes		119 (33.1)		115 (33.3)		121 (34.1)		355 (33.5)							
			No		241 (66.9)		230 (66.7)		234 (65.9)		705 (66.5)							
**Marital status**															0.450			.45
			Single		164 (45.6)		145 (42.0)		146 (41.1)		455 (42.9)							
			Married or living with a partner		196 (54.4)		200 (58.0)		209 (58.9)		605 (57.1)							
**Experienced cancer closely**															0.776			.78
				Yes	263 (73.1)		255 (73.9)		254 (71.5)		772 (72.8)							
				No	97 (26.9)		90 (26.1)		101 (28.5)		288 (27.2)							
**Pharmacy loyalty card**															0.445			.45
		Yes			264 (73.3)		263 (76.2)		256 (72.1)		783 (73.9)							
		No			96 (26.7)		82 (23.8)		99 (27.9)		277 (26.1)							

^a^Chi-square test.

## Results

### Study Sample

**Figure 1:**
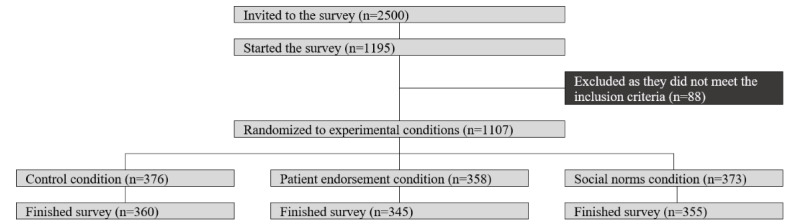
Flow through the study.

Figure 1 demonstrates the flow of participants through the study. In total, 2500 invitations were sent out on behalf of University College London researchers to women registered on a survey panel (Dynata), and 47.8% (1195/2500) responded to the invitation. Out of these potential participants, 92.6% (1107/1195) were eligible for the study.

Eligible participants were randomized to the experimental conditions: 376 to the control condition, 358 to the patient endorsement condition, and 373 to the social norms condition. Across conditions, 4.2% (47/1107) did not finish the survey after randomization, leaving a final sample of 1060, who were all included in the analysis: 34% (360/1060) in the control condition, 32.5% (345/1060) in the patient endorsement condition, and 33.5% (355/1060) in the social norms condition. Most women in the analytical sample were in paid employment (705/1060, 66.5%), were married or cohabiting (605/1060, 57.1%), did not have a college degree (549/1060, 51.2%), owned at least 1 loyalty card from a pharmacy (783/1060, 73.9%), experienced cancer closely (ie, either themselves or with family or close friends) (772/1060, 72.8%), and reported good or excellent health (701/1060, 66.1%). Post hoc comparisons revealed that sociodemographic variables did not vary significantly across the experimental conditions (Table 2).

### Intention to Take Part in CLOCS

Overall, the intention to take part in CLOCS was high, with 60% (636/1060) of women stating that they would probably or definitely participate. Table 3 shows the distribution of intentions after reading the Twitter messages. The ordered logistic regressions in Table S1 in Multimedia Appendix 1 show that the behavioral messages, both patient endorsement (odds ratio [OR] 0.74, 95% CI 0.56-0.98; *P*=.03 and aOR 0.73, 95% CI 0.55-0.97; *P*=.03) and social norms (OR 0.74, 95% CI 0.54-0.93; *P*=.015 and aOR 0.69, 95% CI 0.52-0.92; *P*=.009), decreased intention to take part in CLOCS. None of the sociodemographic variables were significantly associated with the intention to participate in CLOCS.

**Table 3:** Intention to take part in the case-control study.

	Control (n=360), n (%)	Patient endorsement (n=345), n (%)	Social norms (n=355), n (%)	Overall (N=1060), n (%)
Definitely not^a,b^	24 (6.7)	27 (7.8)	29 (8.2)	80 (7.5)
Probably not^a,b^	101 (28.1)	125 (36.2)	118 (33.2)	344 (32.5)
Probably yes^a,b^	183 (50.8)	146 (42.3)	179 (50.4)	508 (47.9)
Definitely yes^a,b^	52 (14.4)	47 (13.6)	29 (8.2)	128 (12.1)

^a^χ^2^_6_=14.52.

^b^*P*=.02.

### Perception of the Messages

Table 4 shows that most study participants stated that the messages were fairly or very easy to understand (796/1060, 75.1%) and that the aims of the study were very or extremely clearly communicated (594/1060, 56%). However, the ordered logistic regression results in Table S2 in Multimedia Appendix 1 show that individuals in the patient endorsement condition perceived the message as more difficult to understand (OR 0.63, 95% CI 0.47-0.84; *P*=.002 and aOR 0.65, 95% CI 0.48-0.87; *P*=.004) and the study aims as less clear (OR 0.70, 95% CI 0.53-0.92; *P*=.01 and aOR 0.72, 95% CI 0.55-0.95; *P*=.02) than those in the control condition. There were no statistically significant differences in the perceptions of those in the social norms condition and those in the control condition.

**Table 4:** Perception of the messages.

				Control (n=360), n (%)		Patient endorsement (n=345), n (%)		Social norms (n=355), n (%)	Overall (N=1060), n (%)		Chi-square test (*df*)			*P* value	
**Understanding the message**												10.9 (6)			.09
	Very difficult		6 (1.7)		10 (2.9)		8 (2.3)		24 (2.3)						
	Fairly difficult		72 (20.0)		94 (27.3)		74 (20.8)		240 (22.6)						
	Fairly easy		213 (59.2)		196 (56.8)		218 (61.4)		627 (59.2)						
	Very easy		69 (19.2)		45 (13.0)		55 (15.5)		169 (15.9)						
**Communication of study aims**												9.23 (6)			.16
		Not at all clear	9 (2.5)		16 (4.6)		15 (4.2)		15 (3.8)						
		A little clear	139 (38.6)		153 (44.4)		134 (37.8)		426 (40.2)						
		Very clear	145 (40.3)		131 (38.0)		152 (42.8)		428 (40.4)						
		Extremely clear	67 (18.6)		45 (13.0)		54 (15.2)		166 (15.6)						

### Active Interest in CLOCS

Almost half of the study participants (526/1060, 49.6%) indicated that they would like to be redirected to the CLOCS website after the survey. The binary logistic regression in Table S1 in Multimedia Appendix 1 shows that participants who were presented with the social norms message were less interested in being redirected than those in the control condition (157/355, 44.2% vs 191/360, 53.1%; OR 0.70, 95% CI 0.52-0.94; *P*=.02 and aOR 0.74, 95% CI 0.54-0.99; *P*=.05). While there were no significant differences between the patient endorsement and control conditions (178/345, 51.6% vs 191/360, 53.1%; OR 0.94, 95% CI 0.70-1.27; *P*=.70 and aOR 0.96, 95% CI 0.71-1.30; *P*=.78), women with a loyalty card (117/205, 52.4% vs 58/144, 40.3%; aOR 1.47, 95% CI 1.10-1.95; *P*=.008), excellent health (70/135, 51.8% vs 22/57, 38.6%; aOR 1.94, 95% CI 1.00-3.76; *P*=.05), aged between 55 and 70 years (117/205, 57.1% vs n/N, 40.3%; aOR 1.89, 95% CI 1.19-3.00; *P*=.007) and those who had experienced cancer closely (404/772, 52.34% vs 122/288, 42.4%; aOR 1.37, 95% CI 1.03-1.83; *P*=.03) were more interested in visiting the study website. Those who had previously participated in health research were less likely to want to be redirected (348/734, 47.4% vs 178/326, 54.6%; aOR 0.75, 95% CI 0.58-0.99; *P*=.04).

### Feedback Question

Table 5 shows the main themes of the content analysis per message. While 57.5% (609/1060) of the study participants were willing to provide some feedback, the majority (395/609, 64.8%) stated that the messages did not require changes. Another common theme was clarity (75/609, 12.3%), where participants thought there was too much jargon, that the message should be shorter, adding the hashtags at the end would make it more readable, and message format (59/609, 9.7%), where participants recommended using brighter colors or adding more infographics or a video instead of text.

Moreover, some participants (237/609, 3.8%) stated that the messages were unclear on how the advertised study uses loyalty cards to help with an ovarian cancer diagnosis. To increase the credibility of the message, 3.3% (20/609) of participants suggested including the university’s or sponsor’s logo at the beginning of the message. Some participants also suggested posting the messages on several social media platforms (16/609, 2.6%), as well as using patient or celebrity endorsement (9/609, 1.5%) or advertising an incentive (7/609, 1.1%).

**Table 5:** Themes extracted from the content analysis for each of the messages.

Themes	Control (n=205), n (%)	Patient endorsement (n=203), n (%)	Social norms (n=201), n (%)	Overall (N=609), n (%)
1. No change	144 (70.2)	118 (58.1)	133 (66.2)	395 (64.9)
2. Clarity of the message	20 (9.8)	32 (15.8)	23 (11.4)	75 (12.3)
3. Format of the message	16 (7.8)	28 (13.8)	15 (7.5)	59 (9.7)
4. Confusion about the advertised study	9 (4.4)	6 (3)	8 (4)	23 (3.8)
5. Credibility of the message	7 (3.4)	8 (3.9)	5 (2.5)	20 (3.3)
6. Advertise on several social media platforms	6 (2.9)	7 (3.4)	3 (1.5)	16 (2.6)
7. Endorsement of patients or celebrity	2 (1)	1 (0.5)	6 (3)	9 (1.5)
8. Advertise an incentive	1 (0.5)	1 (0.5)	5 (2.5)	7 (1.1)
9. Random irrelevant comment	0 (0)	2 (1)	3 (1.5)	5 (0.8)

## Discussion

### Overview

This randomized web-based experiment examined the effectiveness of adding behavioral messages to Twitter advertisements for participant recruitment in a real-world case-control study (CLOCS). The results show that the standard messages yielded the highest intentions compared to the 2 normative behavioral messages. Furthermore, the social norms message decreased the willingness to visit the real study website after the survey. The vast majority of participants stated that the messages did not require changes, but some preferred clearer and shorter advertisements.

### Comparison With Previous Literature

Our findings contrast with previous research, which has shown that using behavioral messages, such as social norms [34,35] and patient endorsement [31,32] can be effective methods to engage with the public on the web. The negative effect we found can partially be explained by the reduced readability of the messages, as individuals in the social norms condition perceived the message to be more difficult to understand—and the aims of the study were less clear than those in the standard advertisement.

Individuals who have had experience with a cancer diagnosis, either themselves, with family, or with close friends, were found to be more interested in CLOCS. This is in line with research reporting that familial history of cancer is associated with increased breast and ovarian cancer screenings due to individuals’ increased awareness of cancer-related complications [44]. In this study, participants’ awareness of ovarian cancer and its risks—partially informed by their close experiences with cancer—may explain their increased interest in CLOCS. Therefore, having a personal awareness of or connection to the proposed project can increase individuals’ interest in health-related research. We also found that women aged between 55 and 70 years had increased interest in visiting the CLOCS website. Ovarian cancer is rare in women younger than 30 years, but the risk increases with age, drastically spiking after 50 years—with the average age of diagnosis being between the ages of 50 and 70 years [45]. Thus, the saliency of the risk for ovarian cancer in these age groups may, in part, explain their interest. Final, while individuals recommended including a video in the message, a recent experimental study did not find any effect of adding animated decision aids to a website with the intention to participate in a case-control study [42].

### Strengths and Limitations

One strength of this study was the use of a randomized experimental design to evaluate the effectiveness of adding behavioral messages to Twitter messages. Additionally, the study used validated questions on intentions and active interest. A final strength of this study is that the statistical analysis included a large number of covariates known to influence participation in health research.

This study has some important limitations, which call for follow-up research. First, the 2 messages were grounded in social norms and patient endorsement, which have mixed and limited evidence supporting the efficacy of these messages in influencing participation in clinical research [46] and may not have been the right theoretical basis for the content of the recruitment messages. This limitation is further exacerbated by the paucity of experimental research testing and reporting different messages on digital and social media platforms and their effectiveness on research recruitment. More theory-based formative research using social media marketing techniques, field experiments, and co-design approaches is needed to improve our understanding of the evidence-based application of social influence on research participation for recruiting participants to health research using social media.

Second, throughout the design and testing of both the social norms and patient endorsement messages, the authors considered whether the messages were suboptimally designed despite having contributions from patient representatives who reviewed the messages, and these have undergone various iterations. This is due to 2 reasons. While previous studies have shown that proximal social norms are more effective in different contexts [30-36], it was not possible to use them in our experiment due to the lack of data supporting the claim at the time of the CLOCS recruitment [37] and the novelty of this case-control study. Additionally, the social norm message had to use a vague verbal quantifier, “many women,” and focus on satisfaction with participation rather than the participation rate to ensure messages were ethical and not coercive. Similarly, the patient endorsement message may not have highlighted the link between motivation and action because the message only referred to the patient representative buying medication for symptoms from retailers before a cancer diagnosis. It is possible that future studies focusing on barriers and facilitators of health research participation in the design of the recruitment messages rather than normative behaviors may demonstrate different outcomes.

The authors aimed to address the aforementioned issues with feedback from the participants. However, this exercise did not lead to clear future recommendations other than the use of the factual message used in the control condition. Nevertheless, the outcomes of this experiment informed the recruitment of the CLOCS participants. The authors gained further understanding of the potential limitations of recruiting participants to CLOCS and successfully recruited 249 participants using Facebook advertisements with the control message [47]. The cost per participant recruited was between US $12 and $19, which is comparable to and less than other health-related studies with a targeted population [48]. This hypothetical experimental study demonstrates the importance of testing messages to be used in internet-based recruitment strategies, the potential limitations, and biases, and not relying only on consensus methods. Embedding process evaluations and pre- and postresearch data collection could have a significant impact on the resources allocated to recruitment as well as whether they reach their intended outcomes. Based on the outcomes of the CLOCS study [37], future studies could emphasize how the participation of women without ovarian cancer in the case-control study can help better early diagnosis of ovarian cancer, use their response rates, and further explore why the existing participants took part in this research to develop effective messages.

Furthermore, in line with previous literature, we measured attitudes toward the simulated Twitter message and CLOCS to capture individuals’ potential reactions to the website [42], which has its limitations, as several studies have reported on the intention-behavior gap [49]. As such, motivational interventions are necessary but often not sufficient to change behavior. It is possible that the hypothetical nature of the web-based experiment may have introduced a potential response bias. Similarly, there might have been a social desirability or agreement bias, where study participants tended to overestimate their intentions.

Last, we did not account for participants’ familiarity with Twitter. Our sample may have contained women who were not used to reading messages containing hashtags (Twitter use was not verified). Moreover, while we tried to include a behavioral outcome by including an option for participants to visit the CLOCS website, our experiment did not formally assess the analytics of the website or investigate how the messages influenced click behavior. Final, this study may have been affected by selection bias, as factors shaping computer use (age, gender, socio-economic status, etc) tend to influence the demographics of the sample in web-based studies [50]. For instance, there are usually similar demographic patterns across social media platforms, where users are primarily made up of young, female, and urban individuals [51,52].

### Implications for Policy and Future Research

Our findings suggest that researchers conducting health-related studies should focus on using simple messages for participant recruitment through Twitter. To increase engagement with potential participants through social media, recruitment messages should be easy to read, transparent, and appropriately targeted to an audience that could have experience related to or an interest in the proposed study. Future research could test messages involving social proofing, such as sharing the experiences of study participants. Additionally, messages could be tested in field experiments by controlling the date, time, hashtags, and images used.

### Conclusion

In conclusion, our results indicate that adding behavioral messages containing patient endorsement or social norms to simulated recruitment messages on Twitter decreased participants’ intention to take part in a real-world case-control study. The social norms message also decreased participant interest in visiting the actual study website. These results can be partially explained by difficulties in reading and understanding the message content, with the addition of normative behavioral components. Future research should continue exploring and optimizing methods that can effectively leverage social media platforms for the engagement of potential participants in health-related research.

## Appendix

Multimedia Appendix 1 Supplementary data.

## Abbreviations

aOR: adjusted odds ratio
CLOCS: Cancer Loyalty Card Study
OR: odds ratio

## Data Availability

The data sets generated during and/or analyzed during this study are available from the corresponding author on reasonable request
