# Supplementary material for: Testing Behavioral Messages to Increase Recruitment to Health Research When Embedded Within Social Media Campaigns on Twitter: Web-Based Experimental Study
Source: JMIR Form Res. 2024 Feb 5;8:e48538. doi: 10.2196/48538 (PMC10877493; doi:10.2196/48538)
Supplement: Multimedia Appendix 1 [file formative_v8i1e48538_app1.docx]

# Multimedia Appendix 1

**Figure S1.** Screenshot of the control message on Twitter


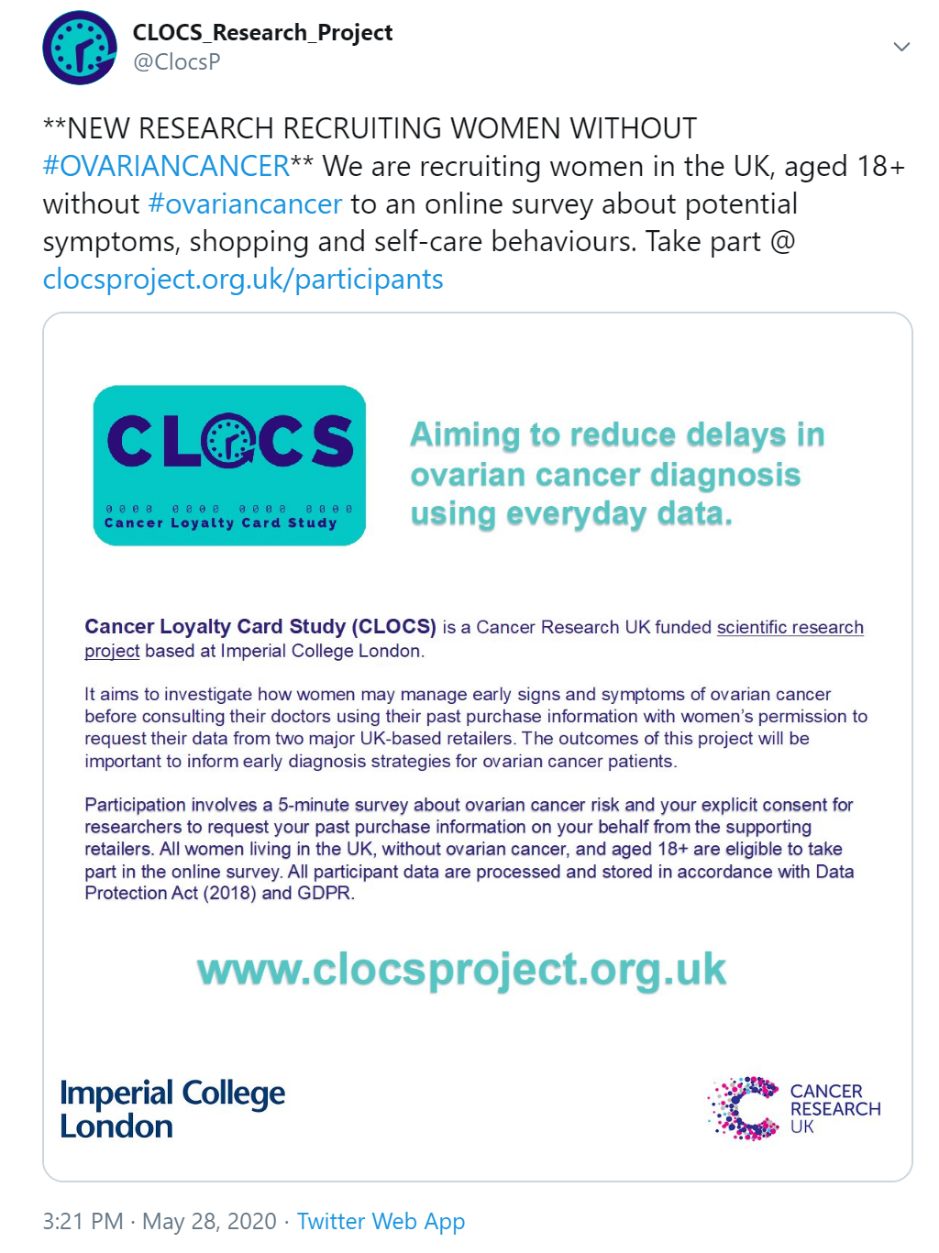


**Figure S2.** Screenshot of the patient endorsement message on Twitter
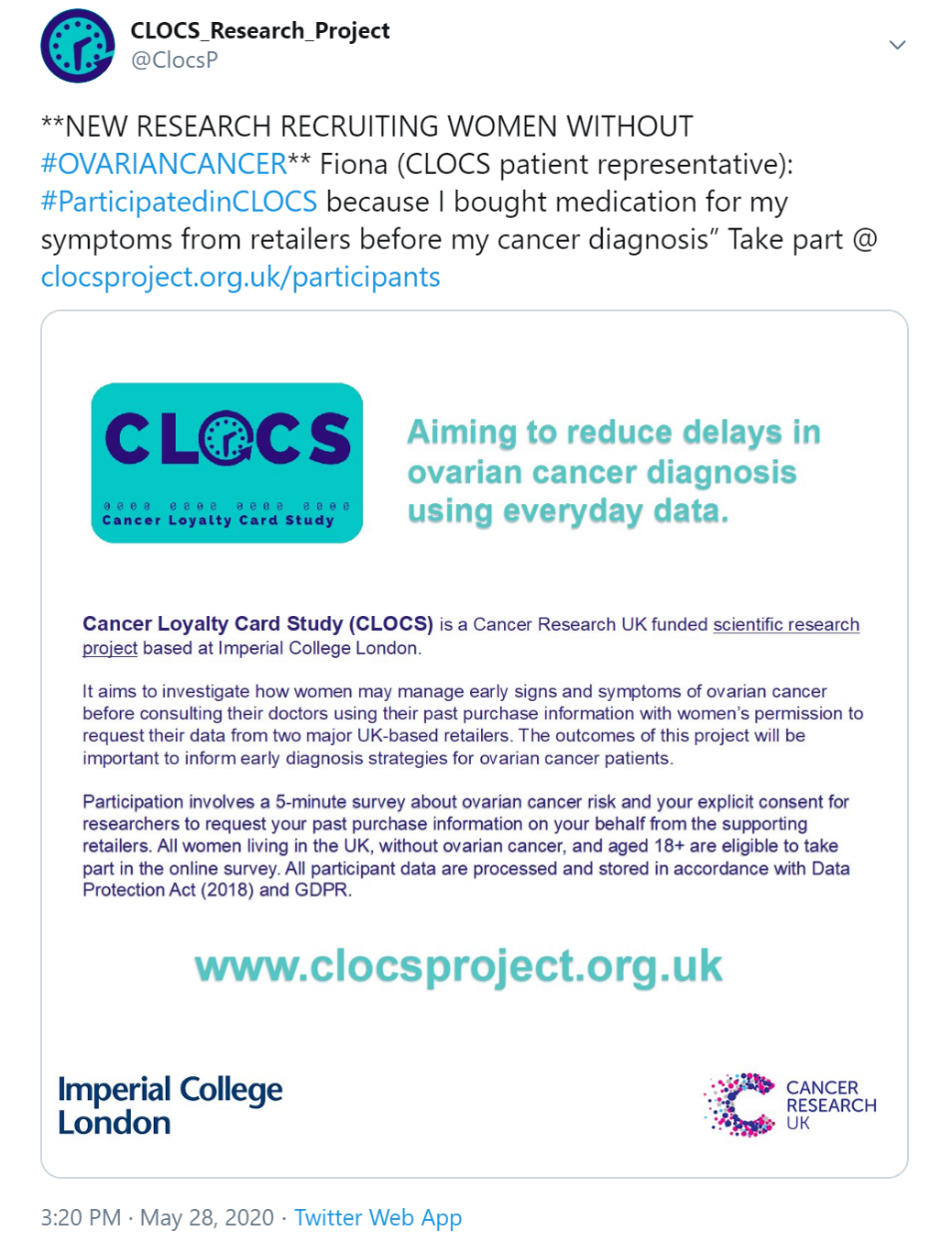


**Figure S3.** Screenshot of the social norms message on Twitter


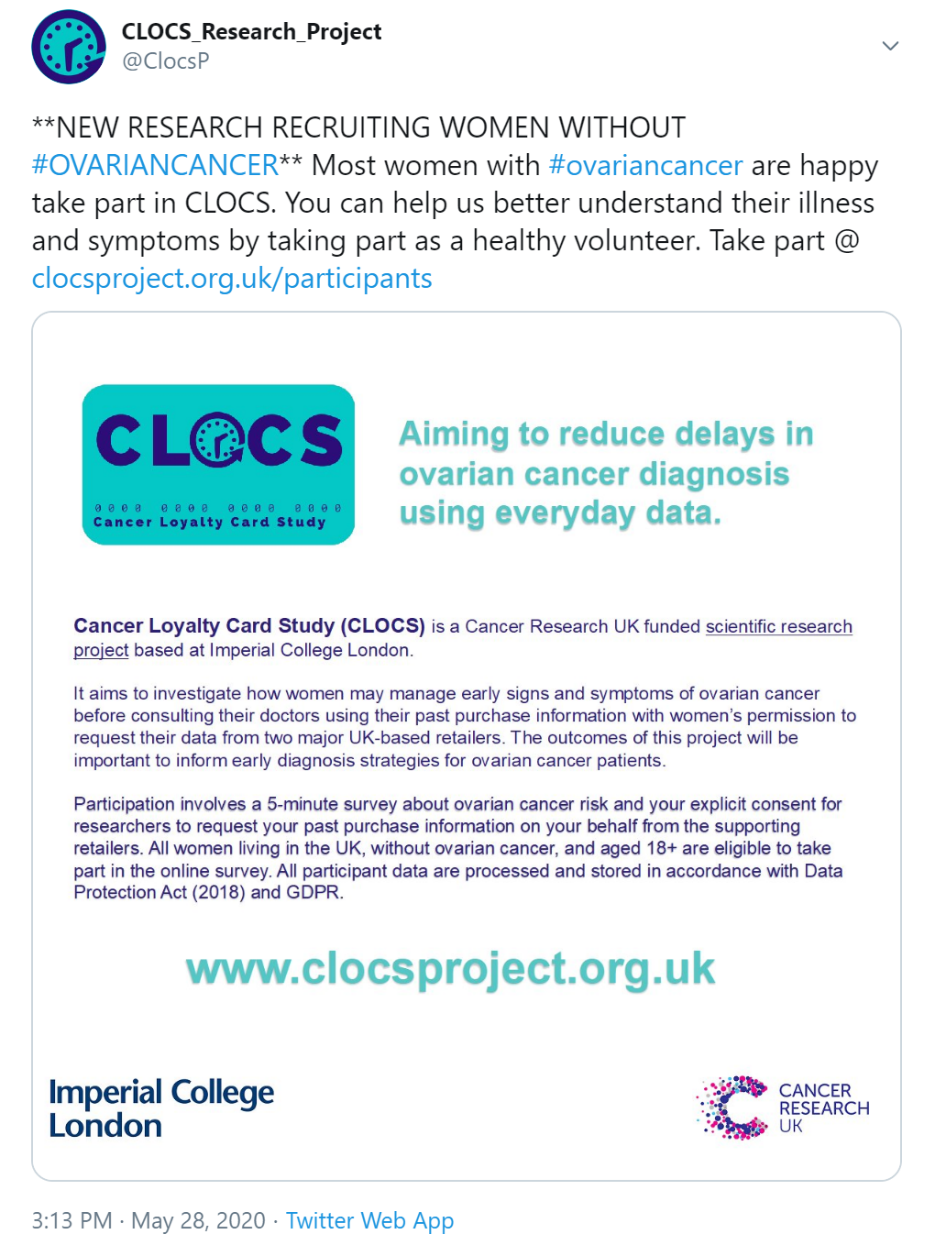


**Table S1.** Ordinal and binary logistic regression on intention to participate in the study and willingness to visit the study website after the survey.

|  |  | Intention to participate in the study [1;4] | | | | | | |  | Willingness to visit the study website [0;1] | | | | | | | |
| --- | --- | --- | --- | --- | --- | --- | --- | --- | --- | --- | --- | --- | --- | --- | --- | --- | --- |
|  |  | Unadjusted regression | | |  | Adjusted regression | | |  | Unadjusted regression | | | |  | Adjusted regression | | |
|  |  | OR^a^ | 95%CI | p-value |  | aOR^b^ | 95%CI | p-value |  | (%) | OR | 95%CI | p-value |  | aOR | 95%CI | p-value |
| Overall |  |  |  |  |  |  |  |  |  | (49.6) |  |  |  |  |  |  |  |
| Condition |  |  |  |  |  |  |  |  |  |  |  |  |  |  |  |  |  |
| Control |  | Ref. |  |  |  | Ref. |  |  |  | (53.1) | Ref. |  |  |  | Ref. |  |  |
| Patient endorsement |  | 0.739 | 0.559 – 0.978 | 0.034 |  | 0.730 | 0.550 – 0.968 | 0.029 |  | (51.6) | 0.943 | 0.702 – 1.268 | 0.698 |  | 0.958 | 0.708 – 1.297 | 0.782 |
| Social norms |  | 0.710 | 0.540 – 0.935 | 0.015 |  | 0.691 | 0.523 – 0.913 | 0.009 |  | (44.2) | 0.703 | 0.523 – 0.942 | 0.018 |  | 0.736 | 0.544 – 0.995 | 0.047 |
| Age |  |  |  |  |  |  |  |  |  |  |  |  |  |  |  |  |  |
| 18-24 |  | Ref. |  |  |  | Ref. |  |  |  | (40.3) | Ref. |  |  |  | Ref. |  |  |
| 25-34 |  | 1.054 | 0.715 – 1.554 | 0.790 |  | 0.984 | 0.653 - 1.483 | 0.939 |  | (49.3) | 1.443 | 0.944 – 2.207 | 0.091 |  | 1.554 | 0.987 - 2.446 | 0.057 |
| 35-44 |  | 1.441 | 0.968 – 2.057 | 0.073 |  | 1.302 | 0.871 - 1.947 | 0.199 |  | (52.6) | 1.643 | 1.086 – 2.487 | 0.019 |  | 1.680 | 1.075 - 2.627 | 0.023 |
| 45-54 |  | 0.992 | 0.677 – 1.451 | 0.965 |  | 0.916 | 0.611 - 1.373 | 0.670 |  | (46.0) | 2.263 | 0.830 – 1.921 | 0.276 |  | 1.253 | 0.799 - 1.967 | 0.326 |
| 55-70 |  | 0.954 | 0.642 – 1.418 | 0.817 |  | 0.834 | 0.547 - 1.273 | 0.401 |  | (57.1) | 1.971 | 1.279 – 3.039 | 0.002 |  | 1.887 | 1.186 - 3.002 | 0.007 |
| Health status |  |  |  |  |  |  |  |  |  |  |  |  |  |  |  |  |  |
| Poor |  | Ref. |  |  |  | Ref. |  |  |  | (38.6) | Ref. |  |  |  | Ref. |  |  |
| Fair |  | 0.995 | 0.588 – 1.684 | 0.984 |  | 0.987 | 0.577 - 1.689 | 0.962 |  | (50.3) | 1.612 | 0.904 – 2.876 | 0.106 |  | 1.624 | 0.892 - 2.955 | 0.113 |
| Good |  | 1.212 | 0.731 – 2.011 | 0.456 |  | 1.160 | 0.688 - 1.956 | 0.578 |  | (49.8) | 1.580 | 0.904 – 2.760 | 0.108 |  | 1.545 | 0.862 - 2.767 | 0.144 |
| Excellent |  | 1.279 | 0.714 – 2.291 | 0.408 |  | 1.284 | 0.704 - 2.341 | 0.415 |  | (51.8) | 1.713 | 0.911 – 3.221 | 0.095 |  | 1.941 | 1.003 - 3.758 | 0.049 |
| Education |  |  |  |  |  |  |  |  |  |  |  |  |  |  |  |  |  |
| Lower than college degree |  | Ref. |  |  |  | Ref. |  |  |  | (49.0) | Ref. |  |  |  | Ref. |  |  |
| College degree, equivalent or higher |  | 1.094 | 0.872 – 1.371 | 0.438 |  | 1.010 | 0.798 - 1.279 | 0.932 |  | (50.3) | 1.053 | 0.828 – 1.340 | 0.673 |  | 0.992 | 0.768 - 1.282 | 0.953 |
| Employment status |  |  |  |  |  |  |  |  |  |  |  |  |  |  |  |  |  |
| Not employed |  | Ref. |  |  |  | Ref. |  |  |  | (47.9) | Ref. |  |  |  | Ref. |  |  |
| Employed |  | 1.106 | 0.871 – 1.404 | 0.407 |  | 0.994 | 0.767 - 1.290 | 0.966 |  | (50.5) | 1.110 | 0.860 – 1.433 | 0.423 |  | 1.096 | 0.824 - 1.457 | 0.529 |
| Marital status |  |  |  |  |  |  |  |  |  |  |  |  |  |  |  |  |  |
| Single/div/widowed |  | Ref. |  |  |  | Ref. |  |  |  | (49.9) | Ref. |  |  |  | Ref. |  |  |
| Married |  | 1.136 | 0.905 – 1.427 | 0.272 |  | 1.138 | 0.892 - 1.453 | 0.298 |  | (49.4) | 0.981 | 0.770 – 1.252 | 0.880 |  | 0.896 | 0.689 - 1.164 | 0.411 |
| Experience cancer close |  |  |  |  |  |  |  |  |  |  |  |  |  |  |  |  |  |
| No |  | Ref. |  |  |  | Ref. |  |  |  | (42.4) | Ref. |  |  |  | Ref. |  |  |
| Yes |  | 1.159 | 0.900 – 1.492 | 0.253 |  | 1.185 | 0.912 - 1.540 | 0.205 |  | (52.3) | 1.494 | 1.137 – 1.963 | 0.004 |  | 1.372 | 1.031 - 1.825 | 0.030 |
| Participated in health research before |  |  |  |  |  |  |  |  |  |  |  |  |  |  |  |  |  |
| No |  | Ref. |  |  |  | Ref. |  |  |  | (54.6) | Ref. |  |  |  | Ref. |  |  |
| Yes |  | 1.123 | 0.880 – 1.433 | 0.353 |  | 1.125 | 0.876 - 1.444 | 0.357 |  | (47.4) | 0.750 | 0.577 -0.974 | 0.031 |  | 0.754 | 0.576 - 0.989 | 0.041 |
| Pharmacy loyalty card |  |  |  |  |  |  |  |  |  |  |  |  |  |  |  |  |  |
| No |  | Ref. |  |  |  | Ref. |  |  |  | (41.9) | Ref. |  |  |  | Ref. |  |  |
| Yes |  | 1.111 | 0.862 – 1.432 | 0.416 |  | 1.101 | 0.851 - 1.424 | 0.465 |  | (52.4) | 1.526 | 1.157 – 2.012 | 0.003 |  | 1.467 | 1.103 - 1.951 | 0.008 |
| *R^2^* |  |  |  |  |  | 0.0086 |  |  |  |  |  |  |  |  | 0.0270 |  |  |
| N |  | 1060 |  |  |  | 1060 |  |  |  |  | 1060 |  |  |  | 1060 |  |  |

^a^OR: odds ratio.

^b^ aOR: adjusted odds ratio

**Table S2.** Ordinal logistic regressions on the perception of the Twitter messages.

|  |  | Difficulty of the message [1;4] | | | | | | |  | Aims of the study were easy to understand [1;4] | | | | | | |
| --- | --- | --- | --- | --- | --- | --- | --- | --- | --- | --- | --- | --- | --- | --- | --- | --- |
|  |  | Unadjusted regression | | |  | Adjusted regression | | |  | Unadjusted regression | | |  | Adjusted regression | | |
|  |  | OR^a^ | 95%CI | p-value |  | aOR^b^ | 95%CI | p-value |  | OR | 95%CI | p-value |  | aOR | 95%CI | p-value |
| Condition |  |  |  |  |  |  |  |  |  |  |  |  |  |  |  |  |
| Control |  | Ref. |  |  |  | Ref. |  |  |  | Ref. |  |  |  | Ref. |  |  |
| Patient endorsement |  | 0.631 | 0.471 - 0.845 | 0.002 |  | 0.646 | 0.481 - 0.868 | 0.004 |  | 0.696 | 0.528 - 0.917 | 0.010 |  | 0.721 | 0.545 - 0.953 | 0.022 |
| Social norms |  | 0.852 | 0.638 - 1.137 | 0.276 |  | 0.852 | 0.636 - 1.141 | 0.282 |  | 0.889 | 0.677 - 1.168 | 0.399 |  | 0.875 | 0.664 - 1.154 | 0.344 |
| Age |  |  |  |  |  |  |  |  |  |  |  |  |  |  |  |  |
| 18-24 |  | Ref. |  |  |  | Ref. |  |  |  | Ref. |  |  |  | Ref. |  |  |
| 25-34 |  | 1.093 | 0.724 – 1.652 | 0.671 |  | 1.089 | 0.705 - 1.682 | 0.701 |  | 1.081 | 0.731 – 1.599 | 0.696 |  | 1.166 | 0.771 - 1.765 | 0.467 |
| 35-44 |  | 1.056 | 0.706 – 1.578 | 0.792 |  | 1.037 | 0.677 - 1.590 | 0.867 |  | 0.945 | 0.645 – 1.383 | 0.771 |  | 1.038 | 0.691 - 1.559 | 0.858 |
| 45-54 |  | 1.212 | 0.806 – 1.822 | 0.357 |  | 1.169 | 0.759 - 1.801 | 0.479 |  | 0.946 | 0.642 – 1.394 | 0.778 |  | 1.031 | 0.682 - 1.556 | 0.886 |
| 55-70 |  | 1.266 | 0.834 – 1.923 | 0.269 |  | 1.225 | 0.786 - 1.911 | 0.370 |  | 0.946 | 0.634 – 1.411 | 0.785 |  | 1.017 | 0.665 - 1.556 | 0.936 |
| Health status |  |  |  |  |  |  |  |  |  |  |  |  |  |  |  |  |
| Poor |  | Ref. |  |  |  | Ref. |  |  |  | Ref. |  |  |  | Ref. |  |  |
| Fair |  | 0.621 | 0.349 – 1.104 | 0.105 |  | 0.658 | 0.364 - 1.189 | 0.166 |  | 0.853 | 0.500 – 1.454 | 0.557 |  | 0.871 | 0.504 - 1.504 | 0.619 |
| Good |  | 0.869 | 0.499 – 1.515 | 0.621 |  | 0.880 | 0.494 - 1.567 | 0.664 |  | 1.202 | 0.719 – 2.007 | 0.483 |  | 1.223 | 0.719 - 2.082 | 0.458 |
| Excellent |  | 1.148 | 0.610 – 2.158 | 0.669 |  | 1.186 | 0.616 - 2.282 | 0.609 |  | 2.036 | 1.133 – 3.659 | 0.017 |  | 2.063 | 1.127 - 3.779 | 0.019 |
| Education |  |  |  |  |  |  |  |  |  |  |  |  |  |  |  |  |
| Lower than college degree |  | Ref. |  |  |  | Ref. |  |  |  | Ref. |  |  |  | Ref. |  |  |
| College degree, equivalent or higher |  | 1.002 | 0.791 – 1.270 | 0.986 |  | 0.945 | 0.737 - 1.211 | 0.654 |  | 0.959 | 0.767 – 1.201 | 0..717 |  | 0.889 | 0.703 - 1.125 | 0.328 |
| Employment status |  |  |  |  |  |  |  |  |  |  |  |  |  |  |  |  |
| Not employed |  | Ref. |  |  |  | Ref. |  |  |  | Ref. |  |  |  | Ref. |  |  |
| Employed |  | 1.050 | 0.817 – 1.349 | 0.702 |  | 1.026 | 0.780 - 1.349 | 0.854 |  | 1.039 | 0.819 – 1.319 | 0.752 |  | 0.979 | 0.755 - 1.270 | 0.873 |
| Marital status |  |  |  |  |  |  |  |  |  |  |  |  |  |  |  |  |
| Single/div/widowed |  | Ref. |  |  |  | Ref. |  |  |  | Ref. |  |  |  | Ref. |  |  |
| Married |  | 1.024 | 0.806 – 1.301 | 0.847 |  | 1.010 | 0.784 - 1.301 | 0.939 |  | 0.928 | 0.740 – 1.164 | 0.516 |  | 0.918 | 0.722 - 1.168 | 0.487 |
| Experience cancer close |  |  |  |  |  |  |  |  |  |  |  |  |  |  |  |  |
| No |  | Ref. |  |  |  | Ref. |  |  |  | Ref. |  |  |  | Ref. |  |  |
| Yes |  | 1.155 | 0.886 – 1.506 | 0.286 |  | 1.139 | 0.865 - 1.499 | 0.353 |  | 0.993 | 0.773 – 1.276 | 0.959 |  | 1.049 | 0.809 - 1.360 | 0.717 |
| Participated in health research before |  |  |  |  |  |  |  |  |  |  |  |  |  |  |  |  |
| No |  | Ref. |  |  |  | Ref. |  |  |  | Ref. |  |  |  | Ref. |  |  |
| Yes |  | 1.222 | 0.945 – 1.579 | 0.126 |  | 1.266 | 0.973 - 1.646 | 0.079 |  | 1.180 | 0.925 – 1.505 | 0.182 |  | 1.183 | 0.923 - 1.517 | 0.184 |
| Pharmacy loyalty card |  |  |  |  |  |  |  |  |  |  |  |  |  |  |  |  |
| No |  | Ref. |  |  |  | Ref. |  |  |  | Ref. |  |  |  | Ref. |  |  |
| Yes |  | 1.266 | 0.963 – 1.662 | 0.091 |  | 1.234 | 0.934 - 1.630 | 0.138 |  | 0.920 | 0.713 – 1.187 | 0.521 |  | 0.942 | 0.727 - 1.222 | 0.655 |
| *R^2^* |  |  |  |  |  | 0.0126 |  |  |  |  |  |  |  | 0.0123 |  |  |
| N |  | 1060 |  |  |  | 1060 |  |  |  | 1060 |  |  |  | 1060 |  |  |

^a^OR: odds ratio.

^b^aOR: adjusted odds ratio
